# New products

**DOI:** 10.1155/S1463924600000110

**Published:** 2000


Journal of Automated Methods & Management in Chemistry, Vol. 22, No. 3 (May–June 2000) pp. 93–95

### New products

### Environmental Analysis: Shimadzu HPLC and GC Determination of Plasticizers

Plasticizers are added to many different industrial products in order to improve plasticity, flexibility, elasticity and other material properities. Esters of phthalic acid, short phthalates, are the most frequently used plasticizers and are commonly added to synthetic—mainly PVC-materials, including adhesives and glues.

Their acute toxicity is generally small, although DOP-(Bis-(-ethylhexyl)-phthalate)—which accounts for 80% of all phthalates in common use—is suspected of causing cancer in animals. One of the most important areas for phthalate determination, therefore, is environmental analysis, to ensure that no potentially harmful compounds are released from the processing stream or present in the environment itself.

A fast, reliable, accurate and cost-effective method for the determination of phthalate levels in environmental matrices is therefore required by the range of industries frequently using plasticizers. Such a technique is available from Shimadzu, a world leader in chromatography and spectroscopy, using HPLC and GC detection methods.

Owing to the complexity of matrices, e.g. soil and sludge, a single method of chromatographic analysis will usually not provide a positive identification of the compounds present. The Shimadzu solution is to apply two methods, using HPLC and GC analysis, or to compare the UV spectra of the sample with those of a standard.

Use of the gradient elution mode ensures that analysis time remains short—one recent Shimadzu test of six phthalates using HPLC methods had a total analysis time of just 16 min—and phthalates can be separated by GC within 8 min, using a temperature programme and pressure programming.

Sample pre-treatment is especially useful if phthalates have to be determined from tap or waste water, where the matrix must be removed and the analytes concentrated. In this instance, phthalates are enriched using solid phase extraction by means of an RP-18 phase, where up to one litre of water is applied to an RP-18 cartridge so that analytes become concentrated onto the solid phase. Compounds of interest are then eluted with a suitable solvent after the washing and drying of the solid phase. The eluent is then diluted to a defined volume for analysis.

Both Shimadzu HPLC and GC techniques have been demonstrated to yield consistent and accurate results—recovery rates for phthalates under the conditions described above are between 91 and 103%—as part of a rapid process which requires little manual time and effort.

*For more information contact Shimadzu at Mill Court, Featherstone Road, Wolverton Mill South, Milton Keynes MK12 5RE, UK. Tel.: + 44 (0) 1908 552200; fax: + 44 (0) 1908 552211. e-mail: Sales@Shimadzu.co.uk*

### Practical screening for drug discovery

With the introduction of new ‘pass-back’ software in its Allegro Combo™, Zymark Corporation has extended the benefits of their Allegro system to non-UHTS applications. By allowing individual readers, washers, incubators and other plate handlers to perform multiple assay steps, even very complex assays can be run using relatively few modules.

This is especially good news for customers who are interested in Allegro’s easy reconfigurability, but who are not processing at the ultra-high-throughput level. It makes Allegro economically attractive for laboratories doing as few as 100–200 plates per day, and answers the need for a versatile, easily scalable assay engine. These applications traditionally would have been performed using complicated, less flexible ‘robo-centric’ systems.

The Allegro system is compatible with virtually all conventional assay techniques, e.g. ELISA and SPA, and will also support future innovations in assay chemistry. It is based on robust, proven technology and consists of interlinked modular workstations in a novel assembly-line architecture.

Allegro has been designed to assure forward compatibility with advances in liquid handling and emerging low-volume assay technology, and can be easily reconfigured to meet changing research needs. It incorporates the sophisticated data handling needed to keep up with its considerable output, plus an advanced software architecture that was built from the ground up to set a new industry standard for reliability and ease of use.

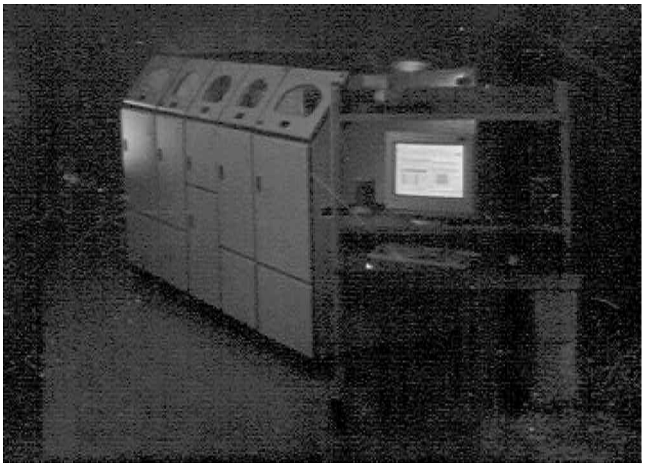

Zymark impacts the world by advancing the ability to discover, develop and market safe and effective drugs that can lengthen and improve the quality of life for everyone. Zymark's employees are active in more than 35 countries and their combined expertise in pharmaceutical analysis and laboratory automation makes Zymark the world's leading supplier in this application area.

*For more information contact: Sharon Correia, Zymark Corporation, Zymark Center, Hopkinton, MA 01748, USA. Tel.: + 1 (508) 497-6403, e-mail: sharon.correia@zymark.com*

### Advanced dual-mode rheometer sets the standard

The new dual-mode CVO120 rheometer from Bohlin Instruments produces accurate and precise viscosity data across a wide range of sample types in either strain- or stress-controlled modes. The instrument's modular design provides complete flexibility for measurement of low-viscosity liquids, viscous fluids and solid materials.

The company has pioneered the use of integrated technology and 'intelligent' software testing to enable meaningful rheological experiments to be performed by both new and inexperienced users.

In combination with other features in the CVO120 package, the 'easy interpretation' data presentation screen ensures that previously complex data can be quantified both quickly and reliably.

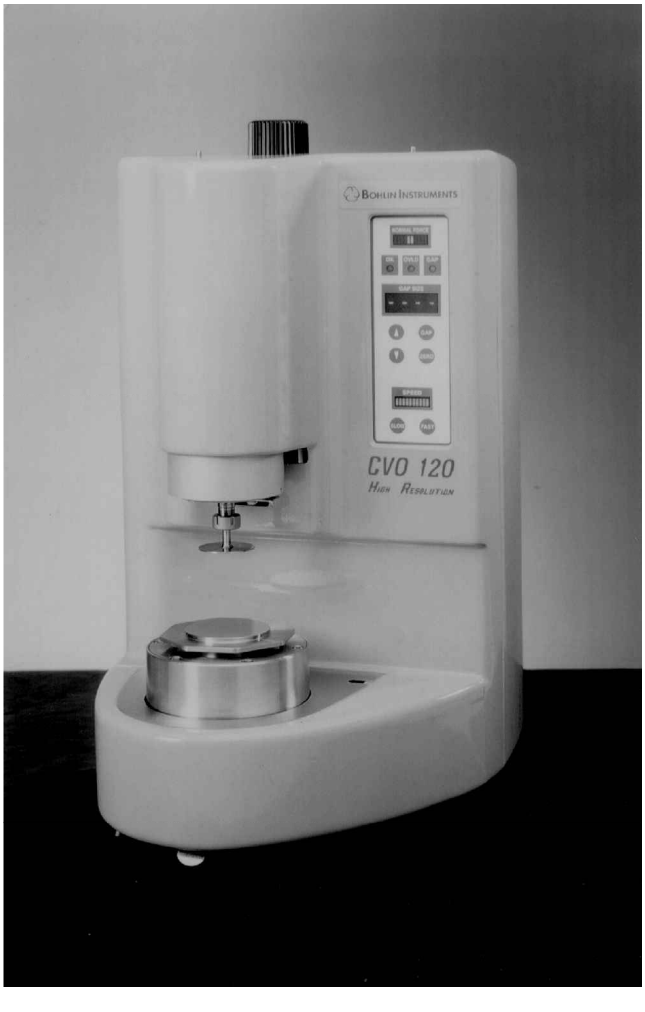

Besides ease of use and extreme sensitivity, the instrument has been designed and engineered as a compact and robust unit. It is thus suitable for both research and industrial testing applications.

The CVO120's dual modes of operation enable it to use either controlled shear stress or shear rate modes to emulate process conditions found in many industrial applications, e.g. mechanical stability of paper coatings, reverse gravure coating of adhesives, spraying of paints, screen printing of inks, etc.

Normal force measurement is also available enabling properties, e.g. extrusion die swell and coating characteristics to be more closely simulated. A specially designed high-temperature cell (range −150–550 °C) provides excellent temperature control allowing samples to be studied in the melt form to provide complete materials characterization.

*For further information, please contact: Martin Aardal, Bohlin Instruments Ltd, Unit 6, The Corinium Centre, Grencester, Gloucestershire GL7 1YJ, UK. Tel.: + 44 (0)1285 644407; Fax.: + 44 (0)1285 644314.*

### Spinning Rifflers—sampling of particulates

Powders and granulates tend to segregate during storage, handling and transportation. Frequently the effect is not visually obvious. Unexplained fluctuations in analytical results, processing requirements, or the final product's properties or yield may be the first manifestation of particlulate segregation at an earlier stage. Research and development work programmes can be delayed or derailed for analogous reasons.

The waste and extra costs caused by unrecognized fluctuations in a material's homogeneity are generally explicable and thus avoidable given the appropriate use of our range of Spinning Rifflers (SRs).

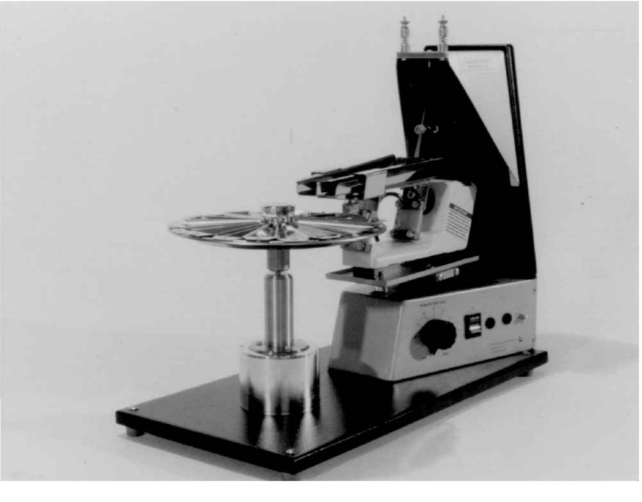

*Micro Spinning Riffler—25 ml capacity*

There are now upwards of 10 SR models and variants, each offering representative powder sampling close to the theoretically attainable accuracy. Starting with the Micro Spinning Riffler (depicted) of 0.0251 capacity, through the 1, 5, 10 and 401 models, this range of instruments has the ability to assist in the research, development and production fields where raw materials, intermediates and final product properties may have a controlling and sometimes critical influence on the final outcome.

*For further information, contact: Charles Templer at Microscal Limited, 79 Southern Row, London W1D 5PL, UK. Tel.: + 44 (0)208 969 3935; Fax.: + 44 (0)208 968 6309.*

### Ninety-six- or 384-well Liquid Handling Workstation

Zymark's Presto™ Liquid Handling Workstation is a powerful new tool for microplate applications. The workstation eliminates the errors and tedium of pipetting into high-density plates. Two pipette arms and individually controlled syringe pumps assure fast, precise liquid transfers. The ultra-small footprint can easily be used on a standard lab benchtop.

The Presto Liquid Handler consistently performs accurate and precise reagent addition, serial dilutions, transfers and plate reformatting using 96- and 384-well plates. The workstation also features exact liquid level sensing and automatic mixing with each liquid delivery step, assuring the uniformity of the assay results.

Presto Liquid Handler software is simple and intuitive with a graphical user interface and point-and-click controls. The Presto Liquid Handler is currently available as a standalone workstation or on integrated assay systems.

Zymark impacts the world by advancing the ability to discover, develop and market safe and effective drugs that can lengthen and improve the quality of life for everyone. Zymark's employees are active in more than 35 countries and their combined expertise in pharmaceutical analysis and laboratory automation makes Zymark the world's leading supplier in this application area.

*For more information contact: Sharon Correia, Director of Marketing Communications, Zymark Corporation, Zymark Center, Hopkinton, MA 01748, USA. Tel.: + 1 (508) 497-6403, e-mail: sharon.correia@zymark.com*

